# Constructing a Prognostic Model for Non-Small-Cell Lung Cancer Risk Based on Genes Characterising the Differentiation of Myeloid-Derived Suppressor Cells

**DOI:** 10.3390/ijms26104679

**Published:** 2025-05-14

**Authors:** Yuheng Tang, Jianyu Pang, Yongzhi Chen, Qi Qi, Hui Wang, Yingjie Sun, Samina Gul, Xuhong Zhou, Wenru Tang

**Affiliations:** Laboratory of Molecular Genetics of Aging & Tumour, Medicine School, Kunming University of Science and Technology, Kunming 650032, China; t1023063288@163.com (Y.T.); jianyu_0898@163.com (J.P.); cyz1206414925@163.com (Y.C.); qiqi12047@163.com (Q.Q.); huiwang266@163.com (H.W.); sunyj6039@163.com (Y.S.); saminagul@kust.edu.cn (S.G.)

**Keywords:** NSCLC, myeloid-derived suppressor cells, tumour immune microenvironment, differentiation, prognosis

## Abstract

Cancer is the most common malignancy, with over 2 million new cases and nearly 1.8 million deaths worldwide annually. Non-small-cell lung cancer (NSCLC) is the predominant subtype, accounting for the majority of cases. Myeloid-derived suppressor cells (MDSCs), which originate from monocytes and typically differentiate into macrophages and granulocytes, possess potent immunosuppressive capabilities. MDSCs regulate immune responses in various pathological conditions and are strongly associated with poor prognosis in cancer patients. This study aims to elucidate the complex interplay between MDSCs, immune cells, and tumours in the NSCLC tumour microenvironment (TME). By integrating single-cell RNA sequencing (scRNA-seq) data with bulk RNA sequencing (Bulk RNA-seq) data, we identified MDSCs as the target cell population and used Monocle software (v2.22.0) to infer their developmental trajectories. We identified key genes associated with MDSCs differentiation processes and classified MDSCs into seven distinct states based on their functional roles. Furthermore, we constructed a prognostic risk model based on the impact of MDSCs differentiation on NSCLC prognosis, utilizing Elastic Net regression and multivariate Cox regression analysis of Bulk RNA-seq data. The model’s performance and accuracy were validated using both internal and external validation sets. Additionally, we compared risk scores with clinical pathological features and the relationship between risk scores and key immune cells in the immune microenvironment, demonstrating the model’s clinical predictive value. We also explored how prognostic genes contribute to poor prognosis in NSCLC. Moreover, small molecule compounds targeting these prognostic genes were screened, and their anti-tumour effects were evaluated as potential therapeutic strategies for NSCLC treatment. This study not only reveals the complex regulatory mechanisms of MDSCs in the NSCLC immune microenvironment but also successfully constructs a prognostic risk model based on MDSCs differentiation states. The model demonstrates excellent clinical performance in predicting patient prognosis, effectively identifying high-risk patients and providing robust support for individualized treatment and immunotherapy decisions. Through association analyses with key immune cells in the immune microenvironment and clinical pathological features, our model can assist clinicians in formulating more precise treatment plans based on patients’ immune status and tumour characteristics. Furthermore, we identified small molecule compounds targeting these prognostic genes, providing novel and promising therapeutic targets for NSCLC, which could further enhance treatment efficacy and improve patients’ survival quality.

## 1. Introduction

According to the World Health Organisation and the International Agency for Research on Cancer, lung cancer is the leading cause of cancer-related mortality, with approximately 350 individuals succumbing to this disease daily. This figure is nearly 2.5 times higher than that of colon cancer, which ranks second in terms of fatality rates. NSCLC constitutes 85% of all diagnosed cases [1]. Current therapies for NSCLC include surgery, radiotherapy, chemotherapy, immunotherapy, molecular targeted therapy, and traditional Chinese medicine. However, the five-year survival rate for patients with this type of cancer remains below 20%. Immunotherapy checkpoint inhibitors have been used as the first line of treatment for advanced NSCLC patients but only show an effective response rate of 63% [2]. Therefore, there is an urgent need for better prognostic tools and more personalized treatment strategies.

MDSCs are derived from monocytes, which serve as precursors for macrophages and granulocytes, capable of undergoing rapid differentiation into mature macrophages, granulocytes, and dendritic cells [3,4]. The role of MDSCs in tumour immune evasion and suppression has been extensively investigated across various cancer types [5]. The prevalence of MDSCs in the peripheral blood and tumour microenvironment of cancer patients has been demonstrated to be increased, which is closely associated with both the disease extent and cancer stage [6]. Not only that, but there is also a strong correlation between the presence of MDSCs and the efficacy of anti-cancer immunotherapy. In various cancer types, including breast, gastrointestinal, and head and neck cancers, researchers have observed an increase in the abundance of MDSCs, as well as upregulation of specific functional markers and enhanced immunosuppressive activity, all of which appear to contribute to immune evasion strategies employed by tumours. MDSCs not only diminish the immune system’s ability to attack by suppressing T-cell activity but also impact the immune homeostasis within the tumour microenvironment through diverse mechanisms such as regulating antigen presentation and secreting inhibitory factors [7,8]. These findings have unveiled the pivotal role of MDSCs in facilitating immune evasion in cancer, garnering significant research attention. However, the intricate and ambiguous relationship between MDSCs and NSCLC remains unresolved, despite notable advancements made in studies pertaining to other types of cancer. In-depth investigations targeting the regulatory mechanisms governing MDSCs in NSCLC and elucidating their precise function within the tumour immune microenvironment (TIME) will enhance our comprehension of this key cellular population’s involvement in NSCLC development. Consequently, this present study aims to unravel the regulatory network of MDSCs within the TIME specifically for NSCLC, thereby offering novel perspectives towards a more profound understanding of immune evasion mechanisms implicated in lung cancer [9]. However, due to the early stage of research on MDSCs, combined with their transient presence in patients, there is a limited range of markers that delineate their biology and subtypes [10]. To address these challenges, we employed the cutting-edge technology of scRNA-seq, which is specifically designed to capture the genetic profile of individual cells, reveal intercellular heterogeneity, and potentially identify distinct subpopulations [11]. To address the limitations of small sample sizes, we integrated two major scRNA-seq datasets. Subsequently, we employed AUCell to assess the activity of distinct MDSCs gene sets and elucidate their diverse functionalities. Furthermore, cellular communication was analysed to comprehend intercellular interactions. Subsequently, an Elastic Net regression algorithm and multivariate Cox regression were utilised to construct a risk-prognostic model, which effectively predicted and validated its clinical applicability. Lastly, based on the prognostic genes employed in the model, their functions and molecular docking were investigated.

## 2. Results

### 2.1. Cell Type Identification

**Figure 1:**
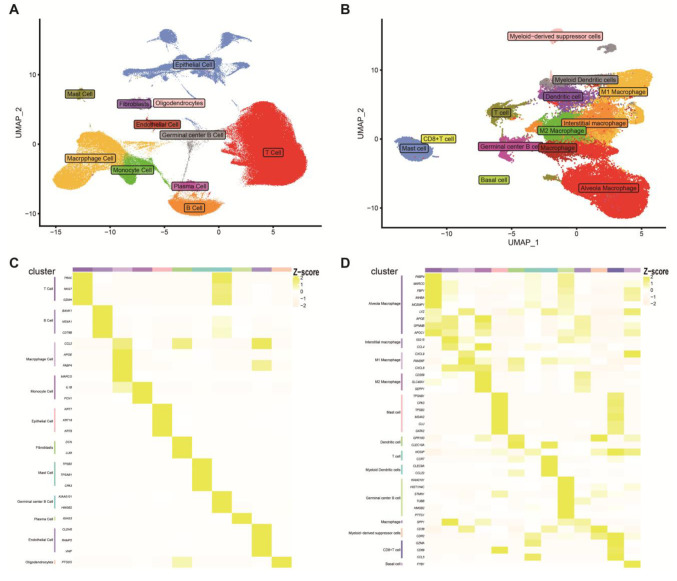
Single-cell analysis: 24 types of cell clusters and their markers were obtained by downscaling and clustering. (**A**) 11 cell types were obtained after the first clustering. (**B**) The second clustering yielded a total of thirteen distinct cell types. (**C**,**D**) Heatmap depicting the gene expression patterns of cell markers. For additional data and detailed methods, please refer to the Supplementary Materials.

This study successfully performed quality control, normalization, dimensionality reduction, and clustering analysis on integrated single-cell data [12], resulting in the identification of 23 distinct cell clusters, which were annotated into 11 cell types, including T cells, B cells, macrophages, and monocytes (Figure 1A). The characteristic expression of these cell types was further validated based on the expression levels of marker genes (Figure 1B). Building on this, we extracted cell populations related to MDSCs, including macrophages, monocytes, and mast cells, and conducted subcluster analysis. This led to the identification of 24 cell clusters, which were annotated into 13 cell types, including MDSCs (Figure 1C). Similarly, the gene expression levels of marker genes for these 24 cell clusters are shown in Figure 1D. Through this detailed cell cluster classification, we were able to identify MDSCs from two GEO datasets for the first time, revealing their critical role in the immune microenvironment.

### 2.2. Distinct Differentiation States of MDSCs in a Proposed Chronology

**Figure 2:**
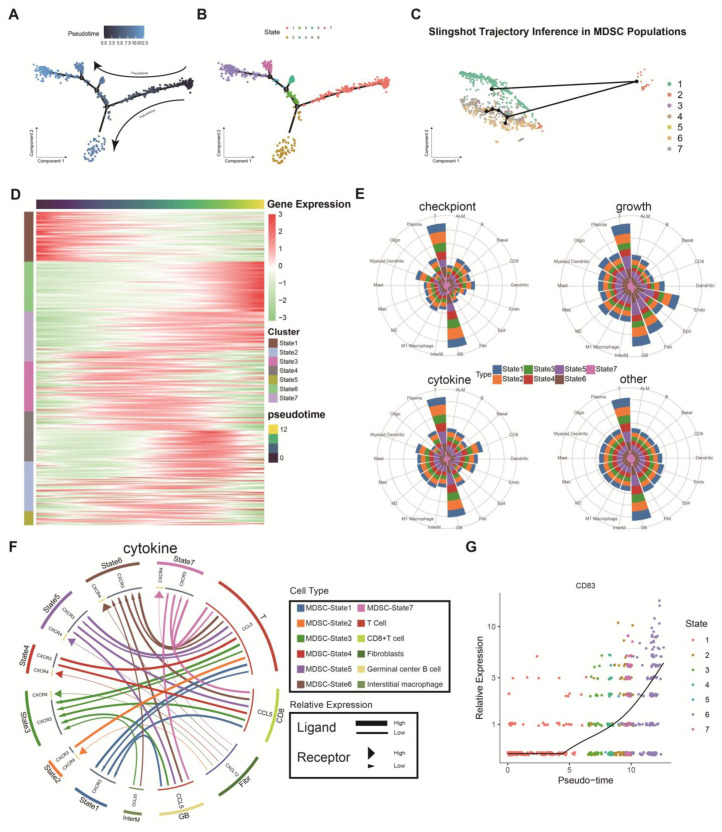
The pseudotemporal analysis of the MDSCs and molecular signalling pathways revealed intercellular interactions. (**A**) The pseudotemporal trajectory of MDSCs inferred by Monocle, illustrating the developmental progression of MDSCs. Branch points labeled as 1, 2, and 3 represent differentiation nodes simulated by pseudotime analysis. (**B**) MDSCs were classified into seven different differentiation states based on their pseudotemporal trajectories. (**C**) Slingshot-predicted trajectory of MDSCs, highlighting the inferred differentiation paths and transition states. (**D**) MDSCs differentiation-related genes (MDRGs) were obtained by differential analysis of differentiation states. (**E**) Radar plot based on distinct molecular mechanisms (such as checkpoints, growth factors, cytokines), illustrating the most prominent interactions between MDSCs and T cells or germinal centre B cells. (**F**) Communication network of MDSCs interacting with other cells. (**G**) Discrimination of two subtypes of MDSCs based on the CD83 marker gene.

To explore the different differentiation states of MDSCs, we employed Monocle to infer and visualize the developmental trajectory of MDSCs and utilised pseudotime analysis to further validate the stability of these trajectories [13]. Although different MDSCs states may coexist within the same tumour sample at a single time point, pseudotime analysis revealed the dynamic evolution of these states, illustrating their sequential relationships during tumour progression. The results (Figure 2A,B) show that MDSCs can be divided into seven distinct states. State 1 predominates in the early pseudotime, and as the time trajectory progresses from right to left, a variety of different states appear in the middle of the pseudotime, with state 6 dominating the late pseudotime. To further validate the inferred trajectories, we applied Slingshot, a trajectory inference tool, to reconstruct the differentiation paths of MDSCs. The Slingshot-predicted trajectory (Figure 2C) confirmed the number and transitions of MDSCs states and highlighted key transitional states, providing additional support for the dynamic evolution of MDSCs during tumour progression. Additionally, we used a gene expression heatmap to display differentially expressed genes between these states (Figure 2D). To investigate the similarities and differences between these seven MDSCs states, we constructed a network based on single-cell data that described the interactions between these states. In the radar plot, considering four distinct molecular mechanisms, such as checkpoints, growth factors, cytokines, etc., it was evident that the interaction between MDSCs and T cells or germinal centre B cells was the most prominent (Figure 2E). Furthermore, based on the expression levels of *CD83*, we identified polymorphonuclear MDSCs (PMN-MDSCs) as the major subpopulation in state 1, while the remaining subpopulations were composed of monocytic MDSCs (M-MDSCs) (Figure 2G). The binding of PMN-MDSCs in state 1 to *CXCR3* was associated with cell recruitment and immune modulation in the early tumour microenvironment, whereas in other states, the involvement of *CXCR4* was linked to cell migration, survival, and other immune evasion mechanisms [14] (Figure 2F).

### 2.3. Gene Set Activity Scores for Different MDSCs States

**Figure 3:**
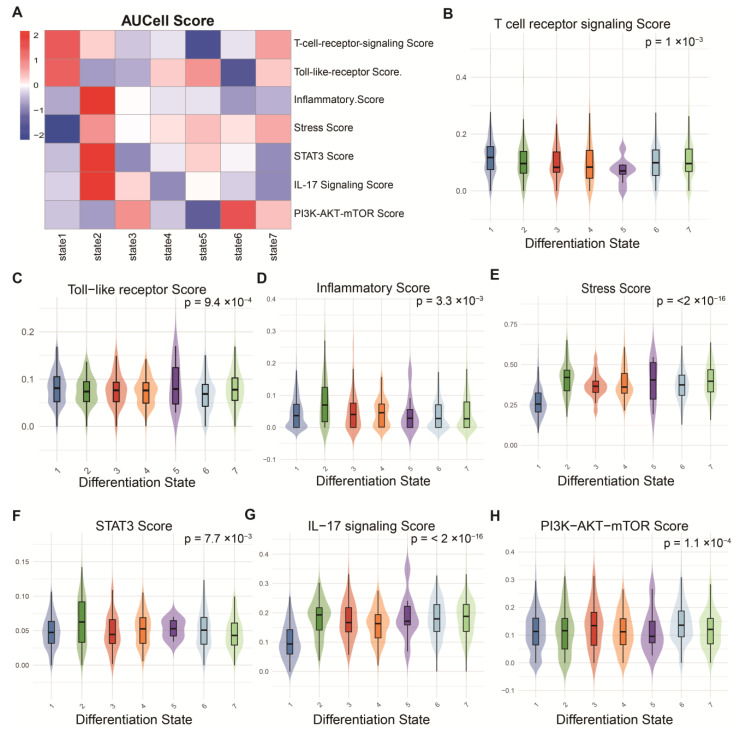
Pathway activity scores of MDSCs in different states. (**A**) Heatmap of pathway activity scores. (**B**–**H**) Analysis of pathway activity differences across states.

In the study of different differentiation states of MDSCs, we found that with the progression of states, the immunosuppressive efficacy of MDSCs gradually increased, playing a key role in tumour immune evasion. In state 1, MDSCs exhibited low immunosuppressive activity, with high scores for T-cell receptor (TCR) signalling and Toll-like receptor pathways, indicating that the immune system could still effectively execute immune responses, which helps suppress tumour growth and metastasis (Figure 3A–C). However, as the tumour microenvironment progressed, the immunosuppressive effects of MDSCs began to enhance. In state 2, excessive accumulation of MDSCs accompanied by the activation of inflammation and the IL-17 pathway suggests that the tumour microenvironment started to promote tumour growth, metastasis, and invasion [15] (Figure 3A,D,F,G). Further analysis revealed that in state 5, low TCR scores and high activity of the PI3K/AKT/mTOR pathway indicated that MDSCs enhanced immunosuppressive effects, aiding tumour immune evasion (Figure 3A,B,H). In state 6, the high expression of the PI3K/AKT/mTOR pathway coupled with low expression of Toll-like receptors may lead to abnormal tumour proliferation and immune escape, thereby limiting the effectiveness of immunotherapy (Figure 3A,C,H) [16].

### 2.4. Identification of Specific Cellular Interactions

**Figure 4:**
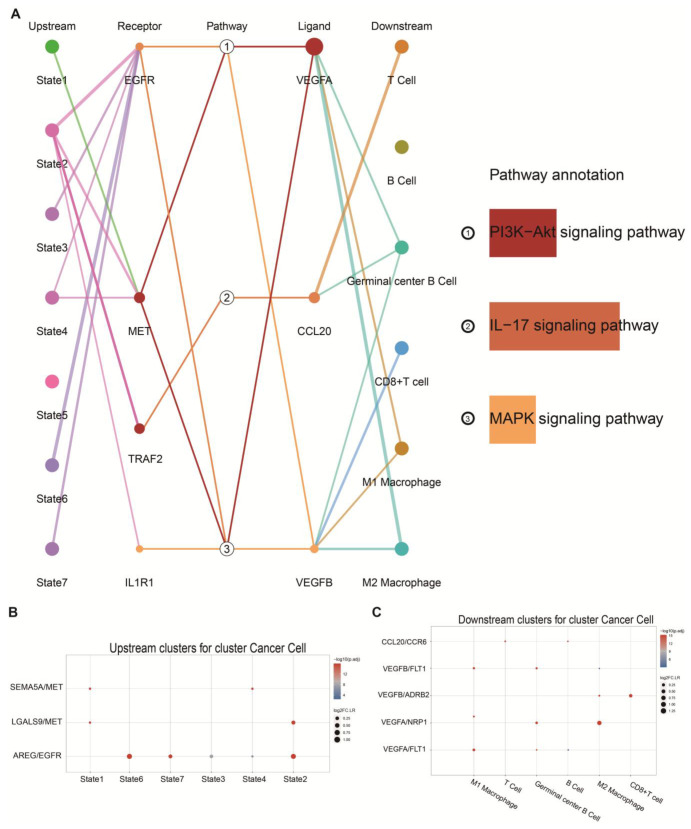
Interaction networks between MDSCs and NSCLC as well as immune cells. (**A**) Cellular communication network between NSCLC cells, MDSCs, and immune cells. (**B**,**C**) Ligand-receptor interaction networks between upstream and downstream.

The cell interaction network analysis revealed that *SEMA5A* ligands on MDSCs in states 1 and 4, as well as *LGALS9* ligands on MDSCs in states 1 and 2, interacted with the *MET* receptor expressed on tumour cells. Furthermore, the *AREG* ligands secreted by MDSCs in states 2, 3, 6, and 7 bound to the EGFR receptor on tumour cells (Figure 4A,B). Additionally, *VEGFA* released by cancer cells exhibited strong interactions with the *FLT1* receptor present on M1 macrophages and follicular centre B cells, while showing diminished affinity towards *FLT1* on M2 macrophages (Figure 4A,C). The VEGFA-FLT1 axis is implicated in processes such as angiogenesis, vascular permeability, and cell migration [17]. These molecular interactions not only activate oncogenic signalling pathways but also lead to the downregulation of MHC class I molecules, reducing the susceptibility of tumour cells to T-cell-mediated cytotoxicity, thus contributing to immune evasion [18]. Moreover, the activation of the PI3K-Akt signalling pathway further promoted the dysregulated survival, uncontrolled proliferation, and apoptosis resistance of cancer cells. Additionally, *GSTP1* ligands produced by MDSCs in state 2 interacted with *TRAF2* receptors on malignant cells, while *CCL20* secreted by tumour cells bound to CCR6 receptors on T cells and germinal centre B cells (Figure 4A,C). These processes are tightly associated with the IL-17 signalling pathway, a complex and highly active network within the TIME. IL-17 signalling is known to activate the nuclear factor kappa-light-chain-enhancer of activated B cells (NF-κB) and mitogen-Activated Protein Kinase (MAPK) pathways, which are involved in tumour progression, aberrant proliferation, and metastasis, contributing to poor clinical outcomes. Moreover, IL-17 induces further *CCL20* production by tumour cells, establishing a detrimental feedback loop that exacerbates immune evasion and tumour progression [19].

### 2.5. Construction of Risk Prognostic Models

**Figure 5:**
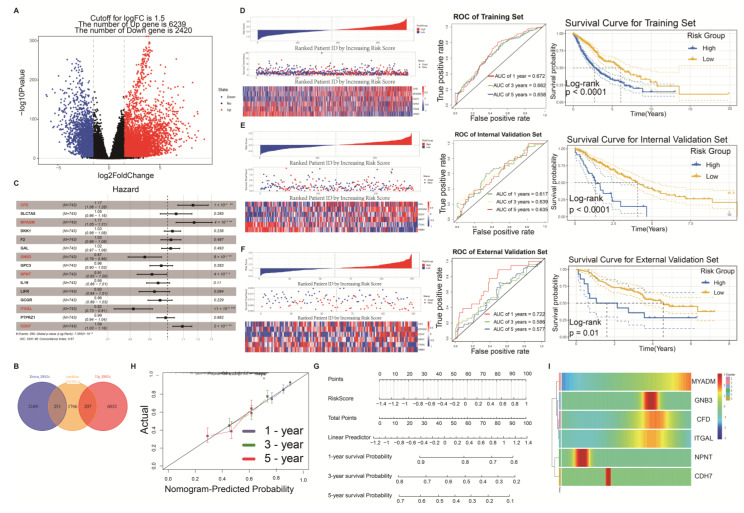
Construction and validation of prognostic risk models. Volcano plot showing differential genes (**A**) and the intersection with MDRGs (**B**). After computation using the Elastic Net algorithm, six genes with prognostic risk model characteristics were selected through multivariate Cox regression analysis. The symbols *, **, and *** indicate statistical significance levels: * *p* < 0.05, ** *p* < 0.01, *** *p* < 0.001 (**C**). (**D**) Displays the risk score distribution, patient status, and prognostic gene expression heatmaps for the training set arranged from low to high based on risk scores. Following this are the ROC curve and KM survival curve. (**E**,**F**) Display the risk score distributions, patient status, and prognostic gene expression heatmaps for the internal validation set (**E**) and external validation set (**F**), arranged from low to high based on risk scores, followed by the ROC curve and KM survival curve. (**G**) Nomogram of the prognostic risk model. (**H**) Calibration curves of the nomogram used to predict 1-, 3-, and 5-year survival. (**I**) Differential expression analysis of prognosis-related genes in different cell differentiation states.

In the construction of the risk prognostic model, we integrated datasets from TCGA-LUAD (*n* = 585) and TCGA-LUSC (*n* = 550) and divided the TCGA cohort into a training set (*n* = 746), an internal validation set (*n* = 264), and an external validation set from the GEO cohort (*n* = 196). To differentiate the differential genes associated with various MDSCs differentiation states, we selected genes that intersected with the complete set of cytokine genes as prognostic genes for the model. The rationale for selecting cytokine genes as key prognostic markers was primarily based on the critical role of MDSCs in tumour immune evasion. MDSCs modulate immune responses in the tumour microenvironment by secreting various immunosuppressive cytokines, inhibiting anti-tumour immunity, and promoting tumour growth and metastasis. Therefore, cytokine genes hold significant biological relevance in this study. Differential expression analysis was performed using DESeq2, which employs a negative binomial distribution to model gene expression data and normalizes the count data across samples. The criteria for differential gene selection first included the application of *p*-value adjustment (padj < 0.01) using the Benjamini–Hochberg false discovery rate (FDR) correction to control for false positives. Second, we adjusted for effect size by filtering genes with |log2FoldChange| > 1.5 to ensure that the selected genes reflected significant biological changes. Subsequently, based on these differentially expressed genes, we further selected cytokines related to MDSCs differentiation states (Figure 5A) and intersected them with other cytokine gene sets from the cancer microenvironment (Figure 5B). This step ensured that the prognostic genes we selected encompassed both cell differentiation characteristics and features of the cancer microenvironment. We constructed the prognostic model using Elastic Net regression and Cox regression analysis. First, from 458 genes, we selected 15 genes with non-zero coefficients through the Elastic Net algorithm. These genes were further analysed using multivariate Cox regression, and six genes with a *p*-value less than 0.01 were identified as the final prognostic genes (Figure 5C). The selected six genes were *CFD*, *MYADM*, *GNB3*, *NPNT*, *ITGAL*, and *CDH7*. Subsequently, the expression levels of these six genes in NSCLC data were multiplied by the corresponding regression coefficients obtained from the Elastic Net model.

The risk score was calculated as follows: Riskscore = 0.1527 × *CFD*_Exp_ + 0.1591 × *MYADM*_Exp_ − 0.1359 × *GNB3*_Exp_ − 0.092 × *NPNT*_Exp_ − 0.204 × *ITGAL* + 0.089 × *CDH7*_Exp_. The final prognostic model was established, which we defined as MDSCs differentiation-related genes (MDRGs). This model can be specifically applied to predict the adverse prognosis of NSCLC influenced by MDSCs differentiation. In the validation set (*n* = 746), patients were stratified into high-risk and low-risk groups based on the median score, illustrating distinct survival outcomes and gene expression patterns between these two groups. Notably, patients in the high-risk group exhibited higher mortality rates and shorter survival times. The Kaplan–Meier survival curve demonstrated superior survival outcomes for patients in the low-risk group compared to those in the high-risk group (*p* < 0.0001), indicating significant prognostic differences among individuals and highlighting the potential utility of risk scores as prognostic indicators. Furthermore, the area under the curve (AUC) values for the one-year, three-year, and five-year predictions exceeded 0.65, underscoring strong predictive performance of our prognostic risk model for NSCLC patient survival (Figure 5D).

To evaluate the accuracy and generalizability of the model, we performed identical calculations for model validation in both the internal validation set (*n* = 264) and the external validation set (*n* = 196) (Figure 5E,F). The validation results demonstrated that the model’s predictive performance in both the internal and external validation sets was consistent with that of the training set, effectively distinguishing between the high-risk and low-risk groups. The survival curves of the two groups showed statistically significant differences (*p* < 0.01), with receiver operating characteristic (ROC) values exceeding 0.6. These findings suggest that our risk score model has good generalizability and can stably predict patient prognosis across different datasets. We generated Nomogram plots to predict signatures (Figure 5G), with RiskScore being the primary determinant. Notably, our calibration analysis revealed a close correspondence between actual survival rates and those predicted by the Nomogram plot for the one-year, three-year, and five-year survival periods (Figure 5H). These findings underscore the excellent predictive performance of our Nomogram. The expression levels of the six prognostic-related genes in MDSCs exhibited significant differences across seven distinct differentiation states (Figure 5I).

### 2.6. Immunoprediction and Clinical Application of Prognostic Risk Model Efficacy

**Figure 6:**
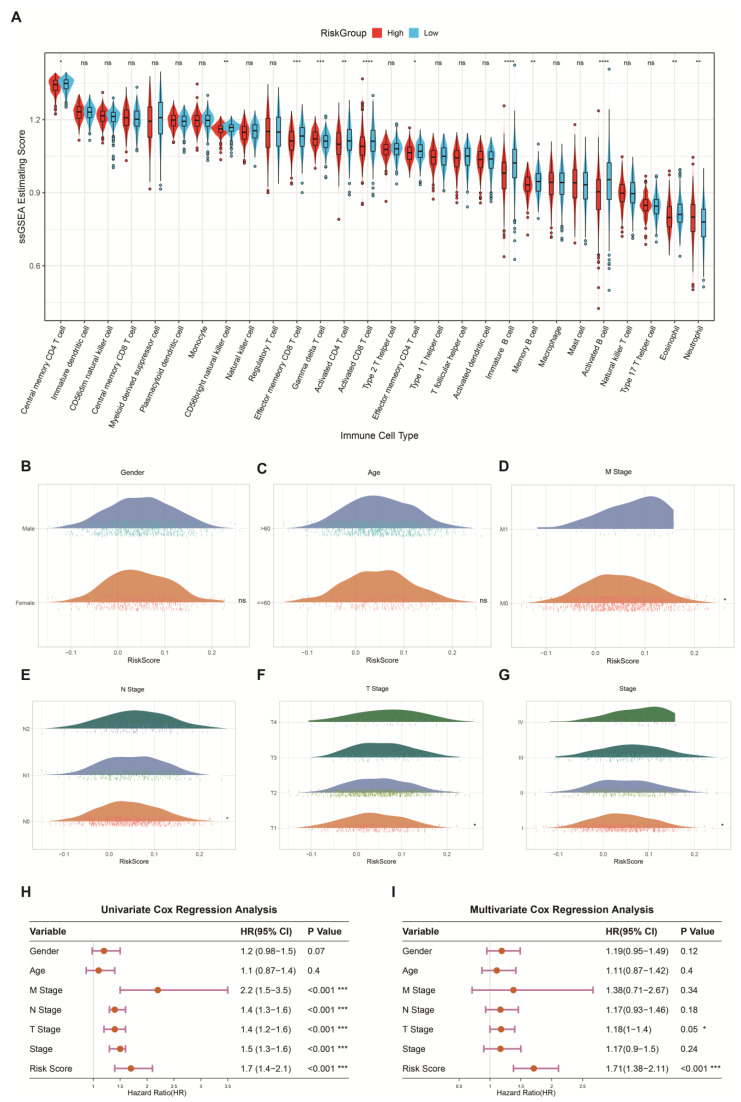
Immune predictive performance and clinical predictive ability of the risk prognostic model. (**A**) Abundance of 28 immune cell types in the high- and low-risk groups. This analysis demonstrates the differences in the abundance of various immune cell types between the high- and low-risk groups.The symbols *, **, ***, ****, and ns represent different levels of statistical significance, where * indicates *p* < 0.05, ** indicates *p* < 0.01, *** indicates *p* < 0.001, **** indicates *p* < 0.0001, and ns indicates *p ≥ 0.05*, with no significant difference. (**B**–**G**) Distribution of clinical features (age, sex, M stage, N stage, T stage, and overall stage) in the high- and low-risk groups. These clinical features were used to assess potential clinical differences between the high- and low-risk groups. (**H**) Univariate Cox regression analysis of the TCGA cohort, exploring the relationship between clinical features and the risk prognostic model. (**I**) Multivariate Cox regression analysis of the TCGA cohort, assessing the combined effect of multiple clinical features on the risk prognostic model.

We conducted a detailed investigation of TIME associated with the prognostic risk score. The single-sample gene set enrichment analysis (ssGSEA) results indicated that 12 types of immune cells exhibited differences between the high- and low-risk groups: Central memory CD4 T cells, CD56bright natural killer cells, Effector memory CD8 T cells, Gamma delta T cells, Activated CD4 T cells, Activated CD8 T cells, Effector memory CD4 T cells, Immature B cells, Memory B cells, Activated B cells, Eosinophils, and Neutrophils. Among these immune cells, the most significant differences were observed in Activated CD8 T cells, Immature B cells, and Activated B cells (*p* < 0.05) (Figure 6A). We observed that the abundance of these three immune cell types was significantly lower in the high-risk group compared to the low-risk group. Activated CD8+ T cells play a crucial role in tumour immune responses, and their reduced abundance may lead to the suppression of anti-tumour immunity. Immature B cells and activated B cells are key players in antibody production, and their reduction could weaken humoral immune responses, further supporting the immunosuppressive characteristics of the immune microenvironment in the high-risk group. Specifically, in the tumour microenvironment of the high-risk group, immune cell function is suppressed, leading to a weakened anti-tumour immune response. Additionally, although other immune cells, such as Effector memory CD8 T cells, Memory B cells, Eosinophils, and Neutrophils, also exhibited some differences, these differences were relatively minor or did not reach statistical significance. Therefore, the immune escape characteristics in the high-risk group primarily resulted from the functional suppression of CD8+ T cells and B cells. This further supports the close association between high-risk scores and immune tolerance as well as tumour immune escape, which may contribute to the formation of a more suppressive tumour microenvironment that promotes tumour growth and metastasis.

Furthermore, we further investigated the relationship between the risk score and various clinical characteristics, including M stage, N stage, T stage, and overall tumour stage. By comparing the risk scores across different clinical stages, we found significant differences in the risk scores among the different M stage, N stage, T stage, and overall tumour stage groups (Figure 6B–G). Notably, as the risk score increased, the severity of stages I, II, and III also increased (Figure 6G), with higher prognostic risk scores being associated with larger primary tumours, greater invasion of surrounding tissues, more extensive overall damage, and poorer prognosis. Next, we performed univariate and multivariate Cox regression analyses to assess the clinical utility of the prognostic model in predicting patient outcomes. Univariate Cox analysis showed that the M stage, N stage, T stage, overall stage, and risk score were significantly associated with the prognosis, with the risk score demonstrating particular significance (*p* < 0.001; HR = 1.7) (Figure 6H), indicating that the high-risk group is associated with poorer prognosis. Furthermore, considering potential confounding factors, we also conducted multivariate Cox regression analysis. The results of the multivariate analysis further confirmed that the risk score is an independent prognostic factor for patients with NSCLC (*p* < 0.001; HR = 1.71) (Figure 6I). These results further consolidate the independent and robust prognostic value of the risk score in NSCLC.

### 2.7. Exploring the Function of Prognostic Genes

**Figure 7:**
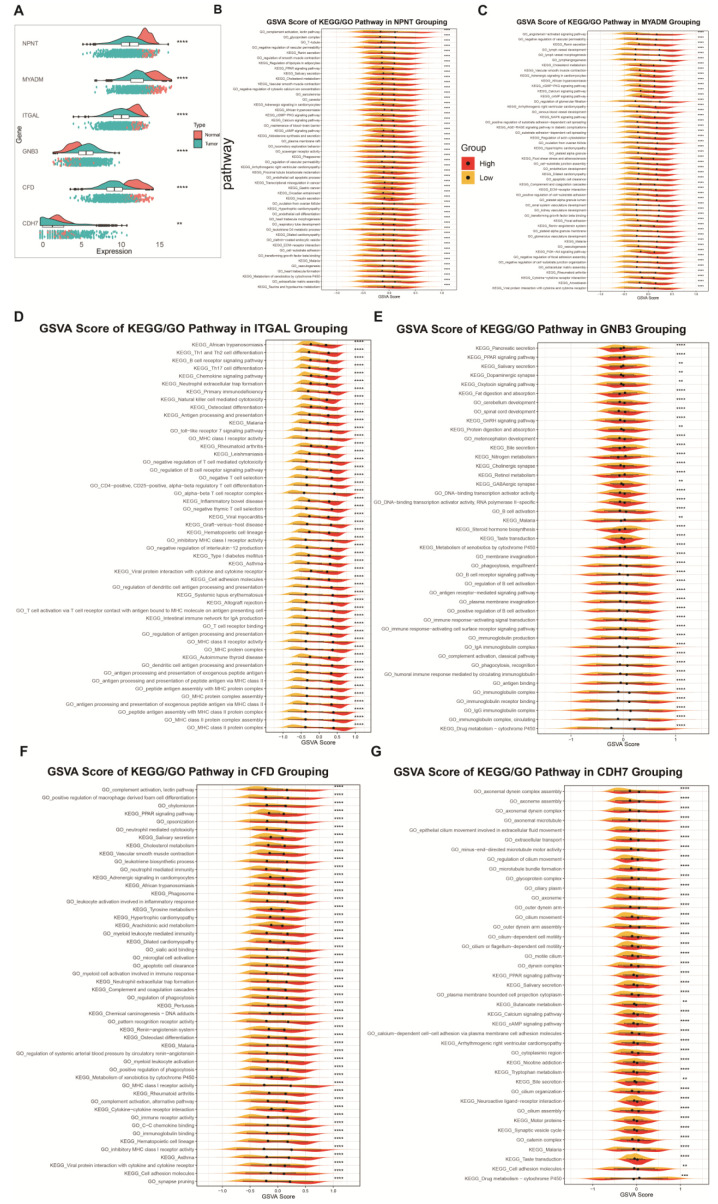
Expression levels, survival analyses and functional studies of six prognostic genes in the TCGA cohort. (**A**) Expression levels of six prognostic genes in the TCGA cohort. (**B**–**G**) GSVA scoring of KEGG and GO pathways for six prognostic genes. The symbols **, ***, and ****, represent different levels of statistical significance, where ** indicates *p* < 0.01, *** indicates *p* < 0.001, **** indicates *p* < 0.0001.

The expression of *NPNT* was significantly downregulated in tumours (Figure 7A), which led to the attenuation of *NPNT*-associated signalling pathways upon the reduction of *NPNT* expression (Figure 7B). Specifically, the decreased activity of the cyclic Guanosine Monophosphate–Protein Kinase G (cGMP-PKG) signalling pathway contributed to uncontrolled lung cancer cell proliferation and differentiation, thereby promoting tumour growth [20]. Moreover, the suppression of the Negative Regulation of Cytosolic Calcium Ion Concentration pathway led to elevated intracellular calcium ion concentrations, triggering a cascade of physiological and signalling abnormalities that ultimately result in poor prognosis. *MYADM* expression was also downregulated in the tumour group (Figure 7A), and the associated pathways were similarly diminished (Figure 7B). The majority of these affected pathways were linked to an unfavourable prognosis (Figure 7C). Additionally, *ITGAL* showed downregulation in tumours (Figure 7A), suggesting a reduction in Th17 cell differentiation activity, which, in turn, impairs anti-tumour immunity. This impairment reduces the immune system’s ability to effectively detect and eliminate cancer cells (Figure 7D) [21]. Both *CFD* and *CDH7* expression levels were observed to be downregulated in the tumour group (Figure 7A). *CFD* encodes complement factor D, a critical protein involved in regulating immune responses, inflammation, cellular lysis, phagocytosis, and other physiological processes [22]. The pathways impacted by *CFD* in NSCLC are primarily inhibitory, influencing cellular proliferation, apoptosis, and invasion. The reduction in *CFD* activity promotes tumourigenesis. Similarly, *CDH7* plays a crucial role in cell–cell interactions, tissue formation, and migration, and its expression is downregulated in tumours. This downregulation may affect inflammatory responses as well as processes such as cellular proliferation, apoptosis, and invasion. The reduced activity of *CDH7* may be associated with tumourigenesis and progression (Figure 7F,G). In contrast, *GNB3* is upregulated in tumours (Figure 7A) and plays a critical role in cellular signalling. Its upregulation may negatively affect multiple signalling pathways (Figure 7E). Additionally, *GNB3* is involved in the activation of intracellular signalling pathways related to cancer progression and metastasis [23].

### 2.8. Screening of Targeted Drugs

**Figure 8:**
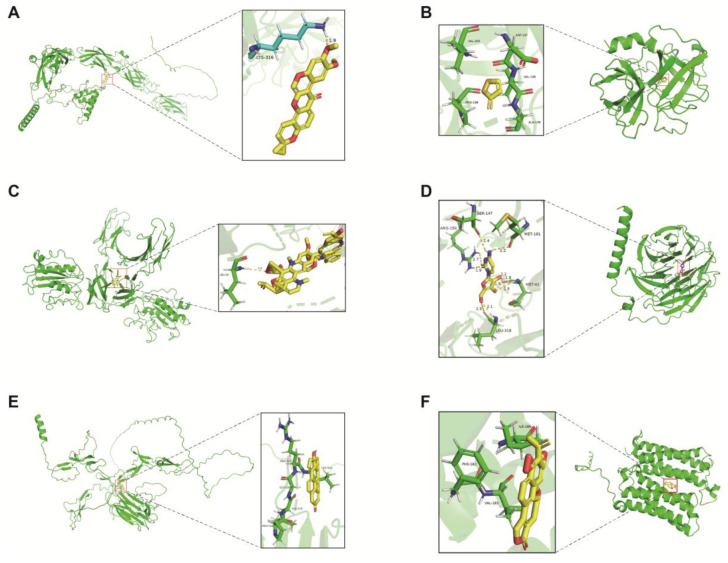
The docking results of proteins encoded by prognostic genes with small molecular compounds. The docking results of deguelin with *CDH7* (**A**). The docking results of 1,2-dithiol-3-thione with *CFD* (**B**). The docking results of vinblastine with *ITGAL* (**C**). The docking results of cytarabine with *GNB3* (**D**). The docking results of methyltestosterone with *NPNT* (**E**). The docking results of triptolide with *MYADM* (**F**).

In our study, Autodock molecular docking was used for targeted drug screening, identifying deguelin as a promising natural compound, which is classified as a soy isoflavone. Deguelin has been shown to inhibit tumour cell proliferation, induce apoptosis, and block angiogenesis [24]. The docking results indicate that deguelin bound to *CDH7* with a simulated binding energy of −1.98 (kcal/mol) (Figure 8A). Additionally, 1,2-dithiol-3-thione, an organosulfide compound with antioxidant, anti-inflammatory, and anticancer properties [25], showed a binding energy of −3.71 (kcal/mol) with *CFD* (Figure 8B) based on the molecular docking results. The optimal docking interaction yielded a binding energy of −3.71 (kcal/mol). Vinblastine, a chemotherapeutic agent belonging to the per-phenanthrene class, is widely used in the treatment of various cancers, including certain types of lung cancer [26]. Our results show that vinblastine exhibited a strong binding affinity with *ITGAL*, with a binding energy of −15.98 (kcal/mol) (Figure 8C). Cytarabine, a nucleoside analogue that closely resembles adenosine, is metabolized in vivo to active metabolites incorporated into DNA strands, thereby disrupting DNA synthesis and inhibiting cancer cell proliferation [27]. Based on molecular docking results, cytarabine showed a simulated binding energy of −5.69 (kcal/mol) with *GNB3* (Figure 8D). Methyltestosterone, a synthetic androgen, showed a simulated binding energy of −4.51 (kcal/mol) with *NPNT* based on molecular docking results (Figure 8E). Triptolide has emerged as an effective anti-lung cancer agent, as even low concentrations significantly suppress cell proliferation, colony formation, and stemness characteristics, while reducing tumourigenicity in sphere-forming cells [28]. Notably, triptolide demonstrated a strong binding affinity for *MYADM* (Figure 8F), with a simulated binding energy value at −6.83 (kcal/mol).

## 3. Discussion

Currently, lung cancer remains the most frequently diagnosed malignancy worldwide and continues to impose a substantial global burden of disease. NSCLC represents the predominant histological subtype. With the continuous advancement of biotechnology, scRNA-seq technology is rapidly evolving, enhancing not only RNA capture efficiency but also enabling the identification of rare cell populations [29]. This technological progress greatly facilitates our investigation into highly heterogeneous tumours. Since Sonenfeld et al.’s discovery of MDSCs in the early 20th century, their potent and specific immunosuppressive capabilities have captivated numerous scientists. Consequently, an array of novel drugs has been developed to target MDSCs with promising prognostic outcomes, rendering them a prominent subject for tumour therapy research [30]. However, due to their relatively short lifespan within the human body and lack of distinctive surface markers, understanding the complex developmental process of MDSCs has been somewhat impeded; thus, it is essential to identify distinct subsets of MDSCs and elucidate their diverse functions.

In this study, cells were partitioned into 20 cell clusters by integrating two large-scale scRNA-seq datasets, following sequential steps of quality control, data standardization, dimensionality reduction clustering, and automatic as well as manual annotation. Within the first cluster, we specifically identified macrophages, monocytes, and mast cells for further analysis in the second cluster. The inclusion of mast cells was based on their relevance to the research question at hand. In an inflammatory milieu, a reciprocal transformation between MDSCs and mast cells has been proposed by certain researchers. Subsequent investigations aimed to elucidate the interrelationship between these two cell types. Utilizing mimetic analysis, MDSCs were subsequently categorized into seven distinct states, which inferred developmental trajectories based on temporal changes in gene expression within different subpopulations of cells. Numerous studies have demonstrated the inhibitory role of MDSCs in immune responses against cancer and other diseases [31]. Investigating and comprehending their mechanisms of differentiation regulation can facilitate the development of more efficacious immunotherapy strategies. Through the results obtained from the interaction network analysis, we gained prior knowledge regarding the T cells that exhibited stronger interactions with MDSCs as well as germinal centre B cells. This not only further validated the accuracy of our cell clustering outcomes but also enabled us to specifically target T cells and germinal centre B cells for subsequent investigations. It is noteworthy that MDSCs themselves possess inhibitory effects on T-cell activity; however, germinal centre B cells also closely interact with T cells, wherein T cells provide signals and assistance to promote the development and differentiation of germinal centre B cells. Moreover, MDSCs influence germinal centre B cell behaviour by affecting T-cell activity, thereby establishing a complex interplay among these cellular components in immune-related diseases or scenarios [32]. The importance of CCL5-mediated signalling in the recruitment of MDSCs has been substantiated by research [33]. However, the accumulation of a limited number of myeloid suppressor cells can facilitate the regulation of immune responses, thereby aiding in the control of tumour growth. Conversely, an excessive accumulation of myeloid suppressor cells contributes to immune evasion by tumour cells. These cells exert immunosuppressive effects that hinder T cell-mediated anti-tumour functions and also contribute to the establishment of an immunosuppressive microenvironment, promoting tumour growth and dissemination [34]. To investigate the seven categories of MDSCs and their distinct functions, we extensively reviewed a substantial body of literature, with a primary focus on the seven signalling pathways that exhibit significant variations in MDSCs. Ultimately, we identified state 1, state 2, state 5, and state 6 as functionally distinct from the remaining states. Based on these diverse functions, it is imperative to develop personalized treatment strategies. Subsequently, through cellular communication analysis, we collected multiple reliable ligand-receptor pairs to elucidate the intricate regulatory network within the tumour microenvironment of NSCLC. Our findings underscored the pivotal role of state II wherein *LGALS9* and *AREG* were found to interact with MET and EGFR expressed by cancer cells, respectively; additionally, *VEGFA* and *VEGFB* expressed by cancer cells interacted with *FLT1* and *NRP1* expressed in M1 macrophages, M2 macrophages, and germinal centre B cells. Furthermore, *CCL20* expressed by cancer cells interacted with *CCR6* on T cells or germinal centre B cells, leading to angiogenesis promotion along with enhanced vascular permeability and cell migration; this interaction also contributed to cancer cell survival enhancement while facilitating evasion from apoptosis. Moreover, it reduces the expression levels of MHC class I gene products, thereby diminishing susceptibility of tumour cells to T-cell attack, ultimately resulting in immune escape from tumours [35]. Although state 5 is not explicitly mentioned in the pathway, our gene set activity scoring analysis reveals that it exhibited downregulated expression of the PI3K/AKT/mTOR pathway, a critical cellular signalling cascade involved in regulating cell growth, differentiation, survival, and metabolism. Activation of phosphoinositide 3-kinase (PI3K) leads to the subsequent activation of protein kinase B (AKT), which ultimately modulates mammalian target of rapamycin (mTOR), thereby exerting influence on diverse biological processes within the cell [36]. The precise regulation of the PI3K/AKT/mTOR pathway is of paramount importance in fields such as cancer research, where aberrant activation of this signalling cascade has been strongly implicated in the initiation and progression of multiple malignancies. In our study, we observed that diminished activity of the PI3K/AKT/mTOR pathway induced by MDSCs in state V led to a reduction in the secretion of immunosuppressive factors, thereby attenuating their inhibitory effect on T cells and compromising the survival and functional potency of MDSCs. This concomitantly resulted in a significant impairment in TCR pathway activity, which, when both pathways were concurrently downregulated, culminated in decreased antigen responsiveness among T cells and consequently exacerbated immune suppression.

Thus far, we have identified functional similarities and differences among seven distinct states of MDSCs. We performed differential gene expression analysis to identify MDSCs’ differentiation-related genes (MDRGs), which were obtained by intersecting the significantly altered gene expression profiles with the complete set of cellular factors. Based on this, we developed a risk-prognostic model comprising six MDRGs, namely *CFD*, *MYADM*, *GNB3*, *NPNT*, *ITGAL*, and *CDH7*.

The expression of arginase 1 (ARG1), an enzyme responsible for the conversion of L-arginine to urea and L-ornithine, has been demonstrated in MDSCs. Importantly, L-ornithine serves as a crucial substrate in the cell cycle process, leading to a significant reduction in extracellular L-arginine levels. A substantial body of research indicates that culturing T cells under low concentrations of arginine leads to functional impairment, primarily due to the downregulation of the CD3ζ subunit of the TCR complex. The CD3ζ subunit plays a pivotal role in facilitating the assembly and activation of the TCR complex [37,38,39] by linking it to downstream signalling cascades through phosphorylation-mediated interactions with immunoreceptor tyrosine-based activation motif (ITAM) motifs on the CD3ζ subunit [40]. Consequently, the depletion of L-arginine reduces the expression levels of CD3-associated ζ chains, thereby inhibiting T-cell proliferation. Simultaneously, [41,42,43]. The protein inducible nitric oxide synthase (iNOS) is exclusively expressed upon stimulation, and its expression strongly correlates with a poor prognosis in malignant tumours. iNOS competes for the same substrates as ARG1, metabolizing L-arginine into citrulline and NO. Nitric oxide (NO) plays a crucial role in tumour progression and serves as a key messenger for T-cell activation. MDSCs can induce immunosuppressive activity by eliminating the IL-2 receptor pathway and nitrosylating the TCR through NO production [44]. Dendritic cells play a pivotal role in bridging the innate and adaptive immune systems, integrating diverse signals from cytokines secreted by innate immune cells to ultimately elicit either responsive or tolerant immune responses [45,46]. Moreover, dendritic cells can acquire tolerogenic properties and exert immunosuppressive effects through modulation of their arginine metabolism. Notably, studies have demonstrated that MDSCs promote the synthesis of ornithine and polyamines via the arginase pathway, leading to upregulation of ARG1 and IDO1 in dendritic cells. This further enhances the TGF-β-triggered immune inhibitory program [47]. Similarly, the chemokine receptor CCR2 expressed by MDSCs and the chemokine CCL2 produced by tumour cells play a pivotal role in mediating the recruitment of MDSCs into tumours; in brief, iNOS in MDSCs hampers T-cell migration through its impact on the chemokine CCL2. Additionally, MDSCs isolated from tumour-bearing mice exhibited significantly elevated levels of reactive oxygen species (ROS) compared to those isolated from normal mice. The biochemical metabolism of MDSCs generates various ROS species, including superoxide (O^2−^), hydrogen peroxide (H_2_O_2_), and peroxynitrite (ONOO^−^). H_2_O_2_ serves as a major contributor to augmenting the pool of ROS. When ROS production was inhibited in MDSCs, their immunosuppressive mechanism was completely reversed as they were able to suppress CD8 T-cell responses via ROS. Similarly, therapeutic strategies targeting MDSCs encompass depletion of these cells, suppression of their immunosuppressive functions, and blockade of their accumulation. For instance, gemcitabine and 5-fluorouracil have been shown to selectively induce apoptosis of MDSCs at both spleen and tumour sites [48]. Carboplatin and paclitaxel effectively reduced abnormally elevated circulating MDSCs and elicited a robust antitumour response in patients with advanced cervical cancer. Gituzumab and ozogamicin enhanced MDSCs mortality, thereby facilitating MDSC depletion in cancer patients. In terms of inhibiting the immunosuppressive function of MDSCs, rectal demonstrated targeted action on MDSCs by significantly suppressing the expression of ARG1, iNOS, and COX2, consequently attenuating the immunosuppressive capacity of MDSCs. Regarding blockade of MDSCs accumulation, the tyrosine kinase inhibitor sunitinib exhibited specific targeting towards MDSCs by blocking VEGF-mediated amplification pathways. Notably, sunitinib significantly diminished MDSCs levels in renal cell carcinoma patients [49].

When evaluating the co-occurrence patterns in human NSCLC, it is crucial to consider the impacts of mutational processes and immune selection [50]. Co-mutations can shape the tumour microenvironment and determine its immune characteristics. The inactivation of the *LKB1* gene has been confirmed in approximately 25% of KRAS-mutant lung adenocarcinomas (LUAD) and has emerged as a primary driver of immune-cold responses. In such cases, NSCLC exhibits a non-T cell inflammatory microenvironment with limited infiltration of CD3+, CD4+, and CD8+ T cells, along with low expression of PD-L1 in tumour cells, despite having a moderate to high tumour mutation burden (TMB) [51]. Moreover, *LKB1* deficiency leads to alterations in the cytokine environment within tumour cells, including increased interleukin expression, thereby promoting bone marrow cell recruitment [52]. Additionally, *LKB1* inactivation induces epigenetic suppression of stimulator of interferon genes (STING), facilitating cytoplasmic dsDNA accumulation that remains insensitive to cellular responses [53]. Finally, loss of *LKB1* also affects the tumour microenvironment in KRAS-mutant mouse models by inducing elevated collagen deposition due to increased lysine levels and influencing angiogenesis [54,55]. In summary, loss of *LKB1* significantly contributes to the immune-cold phenotype observed in NSCLC and may collaborate with *KRAS* through multiple mechanisms during lung cancer pathogenesis.

In our study, MDSCs may have played a significant immunosuppressive role in the high-risk group. MDSCs suppress CD8+ T-cell activation and B cell function by secreting immunosuppressive cytokines, thereby weakening anti-tumour immune responses. We hypothesize that the immune evasion characteristics of the tumour microenvironment in the high-risk group were closely related to the high abundance of MDSCs. MDSCs may play a critical role in immune tolerance and tumour immune evasion, which further contributes to tumour growth and metastasis.

The six genes (*CFD*, *MYADM*, *GNB3*, *NPNT*, *ITGAL*, *CDH7*) utilised in this study to construct a risk prognostic model have been shown in recent studies to be associated with various biological pathways and processes. Specifically, *CFD* has been identified as a rate-limiting enzyme [56] that plays a crucial role in the activation of the alternative complement pathway and innate immune response across multiple cancer types. In addition, *CFD* serves as an independent prognostic biomarker in patients with LUAD and exhibits a significant correlation with clinical outcomes. Our findings demonstrated a substantial downregulation of *CFD* in tumour patients, wherein its effector activity predominantly suppresses pathways that promoted tumourigenesis. Through molecular docking analysis, we identified specific targeted drugs for potential therapeutic interventions. Notably, 1,2-Dithiol-3-thione emerges as a promising candidate due to its antioxidant, anti-inflammatory, and anticancer properties. Previous studies have reported the ability of 1,2-Dithiol-3-thione to modulate carcinogen metabolism in hepatocellular carcinoma by upregulating phase II detoxification enzymes and glutathione levels. *MYADM* belongs to the MAL family and shares certain structural features with other members; however, distinct structural differences still exist [57]. It has been demonstrated that *MYADM* can serve as a prognostic marker in NSCLC [58], Notably, this study reveals the downregulation of *MYADM* in NSCLC patients. Consistent with these findings, low expression levels are often associated with a favourable prognosis in survival curves. The existing literature suggests that *MYADM* is a gene with dual functionality, promoting tumour growth while potentially acting as a suppressor under specific conditions. Furthermore, due to its involvement in complex cellular signalling pathways and regulatory networks, the action of *MYADM* is regulated by multiple factors. Under certain circumstances, reduced expression may contribute to patient survival. Consequently, *MYADM* holds great promise for further research. Triptonide emerges as an effective anti-lung cancer drug capable of significantly inhibiting cell proliferation and colony formation at very low concentrations in lung cancer cells. Additionally, it demonstrates the ability to diminish stemness and tumourigenicity among sphere-forming cells [28]. Furthermore, in the context of prostate cancer, the inhibition of mTOR signalling exhibited remarkable efficacy in suppressing tumour growth and attenuating the activity of associated downstream signalling pathways [59]. *ITGAL*, a member of the integrin family, plays a pivotal role in intercellular adhesion and signalling. Recent studies have reported its association with the TCR signalling pathway, TH17 cell differentiation, and NF-κB signalling pathway. Furthermore, it has been demonstrated that the downregulation of *ITGAL* is not only observed in NSCLC but also serves as a prognostic marker for this disease [60]. This study aimed to elucidate the pathways affected by *ITGAL* downregulation that contribute to poor prognosis in NSCLC patients. Vinblastine belongs to the Vincristine class of chemotherapeutic drugs commonly used for treating various cancers such as lymphoma, testicular cancer, breast cancer, and certain types of lung cancer. Its mechanism of action involves disrupting the microtubule structure within cells, thereby impeding proper mitotic spindle formation during cell division. Consequently, this disruption leads to cell cycle arrest and inhibits proliferation in rapidly dividing cells. Notably, vinblastine has been reported to stimulate an anti-tumour immune response by repolarizing macrophages towards an M1-like phenotype through activation of the NF-κB-Cyba-ROS axis and promoting CD8 T-cell-mediated inhibition of tumour growth [29]. However, it is important to note that while these genes are biologically associated with MDSCs, we must clearly distinguish between genes used to construct the risk prognostic model and those involved in the biological mechanisms of MDSCs. Specifically, genes such as *CFD* and *MYADM* primarily function as prognostic biomarkers, playing a crucial role in predicting the prognosis and clinical outcomes of cancer patients. Their expression levels are closely linked to tumour progression, immune evasion, and immune response regulation, and as such, their role as prognostic genes is more focused on clinical prediction of cancer outcomes. On the other hand, the *ITGAL* gene is more involved in the biological mechanisms of MDSCs, particularly in the TIME, where it plays a critical role [61]. These genes participate in key processes such as immune evasion and immune tolerance, thereby influencing the immunosuppressive state of tumours. Therefore, future research should not only further validate the prognostic value of these genes but also investigate their functions within MDSC-related mechanisms, providing additional therapeutic targets for cancer immunotherapy.

This study aimed to investigate the diverse differentiation states of MDSCs as a novel avenue for comprehensively exploring their similarities and differences. To deeply dissect the intercellular connections, we employed large-scale scRNA-seq, a relatively recent approach for identifying cellular interactions. Grounded in Tumour Immunity in the MicroEnvironment (TIME), this study addressed the intricate interactions within it and further explored prognostic gene functions and targeted drug research. However, this study also has several limitations. In our research, although we performed preliminary functional predictions for six genes (*CFD*, *MYADM*, *GNB3*, *NPNT*, *ITGAL*, *CDH7*) and proposed their potential roles in TIME and MDSCs, we acknowledge that these conclusions are still speculative at this stage. Our study primarily relies on bioinformatics analysis and molecular docking methods, without experimental validation at the protein level or in in vivo models. Therefore, the accuracy and reliability of the findings require further experimental validation. Firstly, regarding the functional predictions of the genes, although the existing literature supports the biological functions of these genes in cancer, we have not directly validated the changes in protein expression or their roles in the tumour microenvironment through wet lab experiments such as protein expression, Western blot, or immunohistochemistry. Future work will validate these findings through the following steps: Initially, we will analyse the expression of these genes using cell line models (such as tumour cell lines or MDSCs cell lines), and utilise techniques like Western blot, qPCR, and immunofluorescence to assess changes in protein and mRNA levels. Next, we will further investigate the effects of these genes on tumour cell proliferation, migration, and invasion using gene knockout or overexpression experiments (clustered regularly interspaced short palindromic repeats-associated protein 9[CRISPR-Cas9] technology or viral vector-mediated gene overexpression). Additionally, to verify the role of these genes in the TIME, we will perform in vivo experiments using animal models (for example, mouse tumour models). We plan to inject tumour cells carrying gene knockouts or overexpressed genes and observe changes in tumour growth and metastasis, coupled with flow cytometry and immunohistochemistry to analyse immune cell infiltration in the tumour microenvironment, particularly focusing on MDSCs alterations. This will help us further understand the relationship between these genes and immune evasion and immune tolerance. In addition to traditional protein expression validation, we also plan to incorporate scRNA-seq and transcriptomics analysis to explore these genes’ roles in the TIME more comprehensively. By isolating and analysing cell subpopulations, we will further refine the interactions between these genes and immune cells, especially MDSCs, and attempt to elucidate their specific mechanisms in tumour immune evasion. In summary, although our study provides preliminary insights into these genes, we acknowledge the speculative nature and limitations of these findings. To enhance the reliability and reproducibility of the research, future work will focus on wet lab validation, particularly in protein expression and in vivo model validation, to further investigate the actual roles of these genes and candidate drugs in the TIME, and to assess their potential as prognostic biomarkers and therapeutic targets.

## 4. Methods

### 4.1. Workflow Diagram of the Study Approach

**Figure 9:**
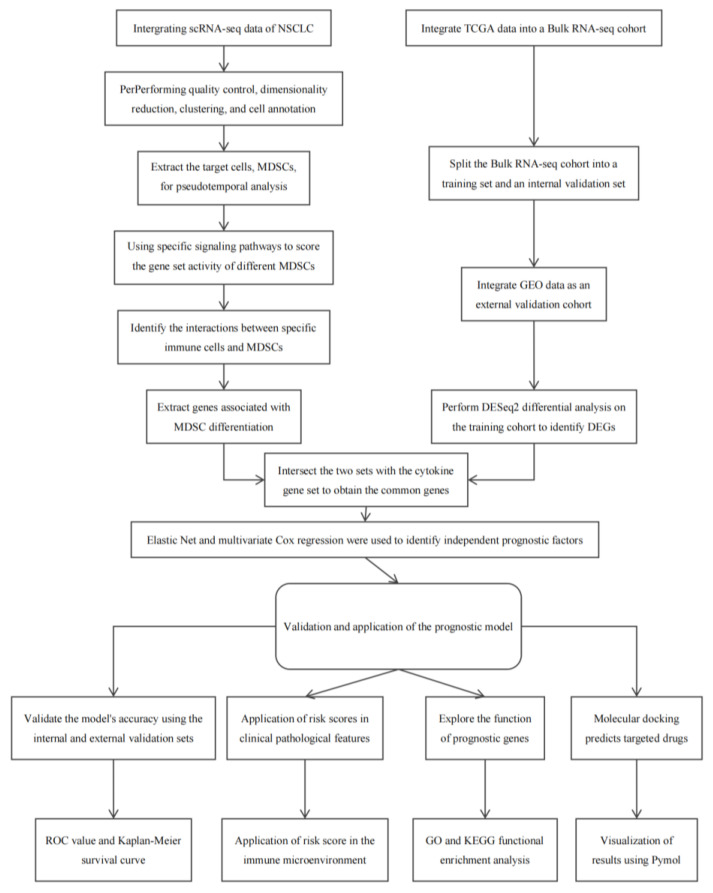
The workflow of the research methods. This diagram illustrates the various steps involved in the study, including sample collection, data preprocessing, signalling network analysis, model construction, and validation.

Research methodology workflow. This schematic outlines the sequential process of the study, encompassing sample acquisition, data cleaning and normalization, signaling pathway exploration, predictive model development, and subsequent validation procedures (Figure 9).

### 4.2. Analytical Data Sources

NSCLC scRNA-seq datasets were downloaded from the GEO database [31], with GSE148071 and GSE162498. The GSE148071 dataset contains data from 42 NSCLC patients, and the GSE162498 dataset contains data from 11 NSCLC patients. TCGA-LUAD (*n* = 585), TCGA-LUSC (*n* = 550), and GSE81089 were derived from the TCGA database [32] and the GEO database. The TCGA cohort was utilised to construct the risk–prognostic model test set and the internal validation set, while the GEO cohort served as the external validation set for the model.

### 4.3. Cellular Annotation

We primarily utilised the Seurat (v4.4.0) package in R for the analysis of scRNA-seq data. Initially, we employed the CreateSeuratObject function to generate a Seurat object that included both the count matrix and metadata for the scRNA-seq data. Subsequently, we performed data normalization using the NormalizeData function to mitigate technical biases across cells, thereby enhancing the accuracy of biological interpretation. Finally, by applying the RunUMAP function, individual cells were projected onto a two-dimensional space, facilitating visualization and cluster analysis. This step aimed to provide a more intuitive representation of the data, thus aiding subsequent interpretation and presentation of results. Upon completion, we systematically employed SingleR (v1.8.1), along with the CellMarker database [33] and the PanglaoDB database [62], for cell-type labelling.

### 4.4. Cell Communication

First, we employed CopyKAT (v1.0.8) to discriminate between cancer cells and normal cells. Next, we utilised the CommPath toolkit for the identification of potential ligand-receptor pairs and conducted statistical analysis on these LR pairs. The threshold of significance level was adjusted to screen LRs (p.thre < 0.01). Simultaneously, pathway enrichment analyses were performed to identify pathways that intersected with LR pairs, and activation scores of pathways in each cell cluster were computed. Through differential activation analysis, we identified significantly upregulated pathways in each cell. Finally, specific cells were selected for bubble plots, network plots, and scatter plots.

### 4.5. Identifying Different Differentiation States of Cells

We employed Monocle (v2.22.0) to construct pseudo-temporal trajectories of MDSCs and annotated the distinct states along these trajectories, as well as identified differentially expressed genes for each state. To further validate the inferred trajectories and capture potential branching events, we applied Slingshot (v1.8.0), a trajectory inference tool, to reconstruct the differentiation paths of MDSCs. Slingshot was used to infer the developmental trajectories and transition states based on the reduced-dimensional representation of the single-cell data, providing additional insights into the dynamic evolution of MDSCs. To comprehend the functional role of MDSCs in various differentiated states, we selected multiple immune-related signalling pathways and retrieved corresponding gene lists from the KEGG database using the KEGGREST (v1.42.0) package to identify key genes within these pathways. In order to evaluate the activity level of these signalling pathways in MDSCs across different differentiation states, we conducted an analysis utilizing the AUCell package and obtained a quantitative measure of pathway activity per individual cell by calculating gene expression levels within each gene set and integrating them at a single-cell level.

### 4.6. Construction of the Prognostic Risk Model

To enhance the predictive accuracy of the model, we utilised Elastic Net regression and multivariate Cox regression methods to construct the prognostic risk model. In Elastic Net regression, we integrated the regularization strategies of Lasso and Ridge regression into the cost function, and adjusted the penalty terms using two parameters, λ and ρ. To select reliable prognostic-related genes, we employed the glmnet package (v4.1-4) in R, with cross-validation to determine the optimal values of λ and ρ. In the gene selection process, differential expression analysis was performed using DESeq2, which employed the negative binomial distribution to model gene expression data. We applied a threshold of padj < 0.01 to control for false positives using the Benjamini-Hochberg FDR correction and further filtered genes with |log2FoldChange| > 1.5 to ensure the biological significance of the results. This approach helped control for false discovery rates while ensuring that the identified genes were biologically relevant. Subsequently, multivariate Cox regression analysis (Cox proportional hazards regression) was conducted to assess the relationship between the selected genes and patient prognosis, constructing the risk score model based on the regression coefficients. To evaluate the predictive accuracy of the prognostic model, we used ROC curves and calculated the area under the curve (AUC) values. Additionally, we constructed a Nomogram to further validate the model’s applicability and clinical utility. All statistical analyses controlled for multiple testing and false positives by adjusting the *p*-values using the Benjamini–Hochberg FDR correction (padj < 0.01). To ensure the robustness of the model, survival analysis was performed to assess the prognostic performance in different risk groups.

### 4.7. Relationship Between Immune Infiltration and Risk Score

SsGSEA is a method for assessing the enrichment level of gene sets in individual samples based on gene expression data. We utilised the ssGSEA algorithm in R to estimate the immune infiltration levels of 28 immune cells in TCGA data. Subsequently, we employed the Spearman rank correlation method to assess the relationship between the risk score and immune infiltration levels, aiming to uncover their potential associations. Finally, to enhance clarity, we visualized these relationships using corrplot, allowing readers to intuitively grasp the relationship between risk scores and immune cell infiltration.

### 4.8. Methodology for Analyzing the Function of Prognostic Genes

The Gene Set Variation Analysis (GSVA) algorithm is a method used for analysing gene expression data to assess the activity level of gene sets within samples. We conducted mutation analysis of prognostic gene sets in the TCGA cohort using the GSVA algorithm in R. This algorithm calculates the GSVA scores for each sample across different pathways, quantifying the activity level of each sample in specific biological pathways. Subsequently, based on the biological grouping results, we identified pathways that exhibited significant activity differences between the high-risk and low-risk groups.

### 4.9. Molecular Docking and Drug Screening

The Autodock tool (Linux, v4.2) was primarily employed for molecular docking and drug screening in this study. Initially, a catalogue of small molecules interacting with prognostic genes was retrieved from the CTD database [63]. Subsequently, structural information regarding these small molecules was obtained from the PubChem database. Furthermore, biomolecules associated with prognostic gene translation were downloaded from the UniProt database [64]. Finally, an automated docking process between biomacromolecules and small molecule compounds was executed according to the designed protocol. To enhance result visualization and analysis, PyMol (v2.6, open source) software was utilised. This carefully devised workflow ensures experiment reproducibility while avoiding redundant data downloads.

## 5. Conclusions

This study characterized seven distinct differentiation states of MDSCs through scRNA-seq, providing preliminary insights into their immune regulatory functions within the tumour microenvironment. Based on gene expression profiles associated with MDSCs differentiation, we developed a prognostic risk model that could predict patient outcomes and assess the potential efficacy of immunotherapy. However, it should be noted that the current conclusions are primarily based on bioinformatics analysis and lack support from wet lab validation (such as validation of protein expression) and in vivo experiments (for example, using mouse models); thus, the accuracy and reliability of the conclusions require further experimental validation. Our study suggests that MDSCs play a crucial role in tumour-mediated immune evasion and highlights the potential of targeting MDSCs-related differentiation genes in cancer immunotherapy. Future work will involve wet lab validation, particularly protein expression, gene knockdown, and in vivo model verification. We expect these follow-up experiments to further advance the development of personalized treatment strategies, ultimately contributing to more effective cancer immunotherapy.

## Data Availability

The datasets analysed in this study are available in GEO and TCGA repositories, including GSE148071, GSE162498, and TCGA-LUAD, TCGA-LUSC. The code used in this study is available upon request by contacting the corresponding author.

## Supplementary Materials

The following supporting information can be downloaded at: https://www.mdpi.com/article/10.3390/ijms26104679/s1.
