# Supplementary material for: Constructing a Prognostic Model for Non-Small-Cell Lung Cancer Risk Based on Genes Characterising the Differentiation of Myeloid-Derived Suppressor Cells
Source: Int J Mol Sci. 2025 May 14;26(10):4679. doi: 10.3390/ijms26104679 (PMC12111218; doi:10.3390/ijms26104679)
Supplement: Supplementary file 1 [file ijms-3594719-supplementary.pdf › ijms-3594719-supplementary.pdf]

For review article

Response to Reviewer 1 Comments

1. Summary
Thank you for giving us the opportunity to revise and resubmit our manuscript titled "Constructing a prognostic model for NSCLC risk based on genes characterising the differentiation of MDSCs." We have carefully considered your comments and suggestions and have supplemented the relevant information accordingly. We hope that everyone will accept our modifications.

**2. Questions for General Evaluation**	**Reviewer's Evaluation**	**Response and Revisions**
Does the introduction provide sufficient background and include all relevant references?	Yes	
Is the research design appropriate?	Yes	
Are the methods adequately described?	Can be improved	
Are the results clearly presented?	Can be improved	
Are the conclusions supported by the results?	Must be improved	

3. Point-by-point response to Comments and Suggestions for Authors
Major points:The gene sets associated with MDSC states lack empirical validation, and there is no functional confirmation of these states or their roles in animal models of disease. To strengthen the findings, the authors should validate the proposed functional states (for proof-of-concept) through in vitro and in vivo experiments for future studies. Additionally, also for future translational purposes exploring and identifying supplementary markers could provide greater resolution in distinguishing MDSC subpopulations, to enhance understanding of the functional heterogeneity of MDSCs found in NSCLC.

Response Major points: *We sincerely appreciate the valuable feedback provided by the reviewers regarding our study. We fully agree with the concern raised about the need for experimental validation of the gene sets and MDSC states. Although we have not yet conducted in vitro and in vivo experiments to validate these states and their roles in disease, this is indeed a major*

research focus for our laboratory. The current study primarily focuses on the development of a predictive model for MDSC states and its potential implications in non-small cell lung cancer (NSCLC), providing a foundation for future research.In our subsequent studies, we plan to experimentally validate the proposed functional states and further explore supplementary biomarkers to better distinguish MDSC subpopulations and deepen our understanding of their functional heterogeneity. Therefore, although this study does not yet include these experimental validations, we have already outlined related follow-up research, and we hope to incorporate these crucial data in the future.

Minor points 1:The figures (1,2, 5, 6, 7) shows detailed visualization (e.g., heatmaps, radar plots) effectively conveys complex data however, they are not clearly legible and of low resolution in the manuscript and its difficult to read of the details.

Response Minor points 1: *We sincerely appreciate the reviewer's valuable feedback on the image quality. In response, we have revised the images for Figures 1, 2, 5, 6, and 7, and have increased their resolution to the highest quality to ensure that the graphical details are clear and visible. Additionally, for the convenience of the reviewers, we have compiled all the modified high-resolution images into a compressed folder and uploaded it. We hope that the updated images will improve readability and better showcase the findings of our study.*

Minor points 2: In results section 2.3 (Gene set activity scores for MDSC states) the authors could include the temporal details about MDSC states. It is unclear as to at what point in tumor progression are these different states found or if they all co-exist together at the same time within the same tumor at given point in time when tumor samples are harvested

Response Minor points 2:*We sincerely appreciate your professional feedback, and we fully*

agree that the temporal details of MDSC states in tumor progression is indeed an important issue that requires further clarification. By incorporating these temporal details, we will be able to more clearly demonstrate the dynamic changes of different MDSC states during tumor progression and their roles in immune evasion.

First, we would like to provide a more detailed explanation of the pseudotime analysis approach. Pseudotime analysis is a method that uses single-cell RNA sequencing (scRNA-seq) data to simulate the temporal order of cells, even if they were not collected in natural temporal sequence.

Second, although these MDSC states co-exist in the tumor samples at the time of collection, our analysis reveals a potential temporal order for these cellular states during tumor progression. Lastly, as mentioned in section 2.2, pseudotime analysis has already been used to define the temporal order of MDSC states in tumor progression (lines 119-123).The results (Figure A-B) show that MDSCs can be divided into seven distinct states. State 1 predominates in the early pseudotime, and as the time trajectory progresses from right to left, a variety of different states appear in the middle of the pseudotime, with state 6 dominating the late pseudotime. This temporal sequence is described in detail in section 2.2.

In section 2.3, we further analyzed the changes in gene set activity across these different MDSC states, highlighting the immune functional differences between them. While different MDSC states may co-exist within the same tumor at the time of sample collection, the pseudotime analysis

provides valuable insights into the dynamic evolution of these states, revealing their sequential relationships during tumor progression.

We hope this clarification addresses the reviewer's concerns regarding the temporal details of MDSC states and further strengthens the depth of our study. Additionally, we have revised the previous results section (lines 114-119) to incorporate these clarifications. Although different MDSC states may coexist within the same tumor sample at a single time point, pseudotime analysis revealed the dynamic evolution of these states, illustrating their sequential relationships during tumor progression.

Minor points 3:Section 2.4 postulates cellular interactions and it demonstrates informative and powerful insights but there is no empirical validation of such interactions in a model system. The authors could demonstrate 1-2 interactions in vitro using cell lines or primary MDSC/Macrophages/Cancer cells as a proof of concept.

Response Minor points 3:*Thank you to the reviewer for their detailed review and suggestions regarding Section 2.4. We highly appreciate the reviewer's comment on the empirical validation of cell interactions. Indeed, experimentally validating these interactions would significantly enhance the credibility of the study. However, as my research is part of a broader project within our research group, our current work mainly focuses on the preliminary stages, and we have not yet reached the phase of experimental validation. Future research will include these experimental validations, particularly using cell lines or primary MDSCs, macrophages, cancer cells, and other relevant models to validate the concept of cell interactions. Therefore, while the current study does not involve these empirical experiments, they are an integral part of our future research plan and will be further explored and incorporated in subsequent studies.*

Minor points 4:Figure 7 an excellent correlation between prognostic genes and the TCGA survival data for patient survival of patients from TCGA

Response Minor points 4:*We would like to thank the reviewer for their detailed review and positive feedback on our study. We are pleased to hear that the reviewer appreciated the correlation between the prognostic genes and patient survival data from TCGA in Figure 7. Indeed, Figure 7 illustrates the strong prognostic ability of the gene model we developed using the TCGA dataset, further validating the model's effectiveness. We believe this finding provides valuable evidence for clinical application and also supports the role of MDSC status in tumor immune evasion. We will continue to explore ways to further validate these prognostic genes and conduct in-depth analyses of their performance in other independent datasets.*

Minor points 5:In Figure 8 the molecular docking analysis identifies promising therapeutic candidates, such as deguelin and triptolide, targeting key prognostic genes however, docking simulations are insufficient to confirm drug efficacy. The authors could substantiate these theoretical findings using in vitro assays to validate the predicted drug effects on NSCLC/MDSC cells.

Response Minor points 5:*We would like to thank the reviewer for their valuable suggestions*

regarding the molecular docking analysis in Figure 8. We fully agree with the reviewer's point that while molecular docking simulations can reveal potential therapeutic drug candidates, they are indeed insufficient to directly confirm the efficacy of the drugs. Currently, our research is primarily focused on the prediction of prognostic genes and the identification of potential targeted drugs,

which is part of a larger project within our research group. As such, we have not yet conducted in vitro validation experiments for these drugs. We plan to carry out efficacy validation of these predicted drugs using NSCLC/MDSC cells in future studies, further confirming these theoretical findings. Therefore, while the current study does not include these in vitro experiments, this work is incorporated into our future research plan, with the goal of providing stronger experimental evidence for translational research.

For review article

Response to Reviewer 2 Comments

1. Summary

Thank you for giving us the opportunity to revise and resubmit our manuscript titled "Constructing a prognostic model for NSCLC risk based on genes characterising the differentiation of MDSCs." We have carefully considered your comments and suggestions and have supplemented the relevant information accordingly. We hope that everyone will accept our modifications.

**2. Questions for General Evaluation**	**Reviewer's Evaluation**	**Response and Revisions**
Does the introduction provide sufficient background and include all relevant references?	Can be improved	
Is the research design appropriate?	Can be improved	
Are the methods adequately described?	Must be improved	
Are the results clearly presented?	Must be improved	
Are the conclusions supported by the results?	Must be improved	

3. Point-by-point response to Comments and Suggestions for Authors

Major points 1:The differentiation trajectory of MDSCs inferred using Monocle lacks robust validation. To strengthen this claim, the authors should incorporate additional computational methods (e.g., alternative trajectory inference tools) and explore the role of related transcription factors in MDSC differentiation. Without such validation, the inferred trajectory remains speculative and unconvincing.

Response Major points 1: *We would like to sincerely thank the reviewer for their feedback on our study. We understand the reviewer's concern regarding the lack of robust validation for the MDSC differentiation trajectory. In our current work, in addition to using Monocle for trajectory inference, we also performed Pseudotime analysis to further validate the stability and biological relevance of these trajectories. Pseudotime analysis is a widely used method for single-cell trajectory inference, where each cell is assigned a "pseudotime" to infer the relative progression of cells transitioning from one state to another. In our analysis, Pseudotime closely aligned with the*

trajectories inferred by Monocle, further supporting the proposed MDSC differentiation process. Specifically, we observed that as pseudotime progressed, MDSCs gradually transitioned from an earlier state to a later state, which was consistent with the results from Monocle, indicating a continuous transition between these differentiation states.

To further validate the biological relevance of the MDSC differentiation trajectory, we incorporated gene sets related to known MDSC-associated signaling pathways in Section 2.3, and performed gene set activity scoring for the seven differentiated MDSC states. This analysis revealed changes in signaling pathway activity across different differentiation states, particularly showing dynamic shifts in certain pathways during the pseudotime process. These shifts were highly consistent with known MDSC differentiation pathways. The gene set activity scoring results provide additional biological support for the proposed differentiation trajectory, further validating its biological relevance. Additionally, we have comprehensively revised the expression of these findings in the main text (lines 154-170).

Major points 2:The connection between gene set scores of different MDSC differentiation states and their immunosuppressive effects is currently speculative and insufficiently supported. The authors should provide more mechanistic evidence, such as experimental data, demonstrating how these gene sets are directly related to MDSC-mediated immunosuppression.

Response Major points 2: *We would like to sincerely thank the reviewer for their valuable feedback on our work. We understand the concern raised regarding the connection between gene set activity scores and the immunosuppressive effects mediated by myeloid-derived suppressor cells (MDSCs), and we would like to address this in more detail.*

In the current analysis, we primarily rely on gene set activity scores to reveal the biological characteristics of MDSCs at different differentiation states, and these scores reflect the activity of known immunosuppressive signaling pathways. However, as the reviewer pointed out, the causal relationship between gene set activity scores and MDSC-mediated immunosuppressive effects indeed lacks direct experimental data to support it.

We acknowledge this limitation and aim to strengthen the credibility of our study and the rationale behind our conclusions through the following points: First, literature support: The gene sets we used are primarily derived from existing studies on MDSC-associated signaling pathways, which have been extensively validated for their role in various immunosuppressive effects. Specifically, pathways such as Toll-like receptors, IL-17, and PI3K/AKT/mTOR have been shown in multiple studies to play critical roles in immunosuppression. To support the association between gene set activity scores and immunosuppressive effects, we will cite relevant literature in the revised manuscript.

Second, biological reasoning: From our gene set activity scoring results, we observed significant changes in the activity of certain immunosuppressive pathways across different MDSC states. While we cannot provide direct experimental data to validate this, these observations are consistent with known mechanisms of MDSC-mediated immunosuppression, suggesting that these pathways

may play important roles in MDSC differentiation and immunosuppressive function.

Lastly, future validation directions: We agree with the reviewer's suggestion for experimental validation and will propose related future research directions in the revised manuscript. We aim to explore how experimental approaches can further validate the association between these gene sets and immunosuppressive effects in MDSCs at different differentiation stages. We will discuss potential experimental strategies, such as using immunohistochemistry, flow cytometry, and other techniques, to investigate the direct roles of specific genes and signaling pathways in MDSC-mediated immunosuppression. We recognize that further experimental validation is required, and our response will provide valuable directions for future research.

We sincerely appreciate the reviewer's insightful comments and look forward to your further feedback.

Major points 3:The differential expression analysis, which identifies 8659 differentially expressed genes (nearly half of all genes), raises serious concerns about the methodology. This large number of differentially expressed genes seems implausible. The authors should clarify their methodology, justify the large number of differentially expressed genes, and consider applying a more selective threshold.

Response Major points 3:*We would like to thank the reviewer for their attention to the number of differentially expressed genes (DEGs). We understand that the 8,659 DEGs may seem excessive, and we would like to clarify our analytical approach. In our study, a large number of DEGs were initially identified because we applied a relatively lenient threshold to ensure that potentially relevant genes associated with MDSC differentiation were not overlooked.*

To refine the analysis and enhance its biological relevance, we performed an intersection analysis with genes previously associated with MDSC differentiation, which significantly narrowed the gene list. This step helped us focus on key genes that are likely to directly participate in MDSC differentiation and immunosuppressive function, ensuring that the genes selected for model construction have higher biological significance.

We have provided a detailed explanation of this process and strengthened the description of this analysis in the revised manuscript. We have clearly outlined why a more lenient threshold was initially chosen and how the subsequent intersection analysis helped refine the gene set.

Once again, we sincerely appreciate the reviewer's careful examination of our methodology. Your suggestions have greatly contributed to improving the transparency and accuracy of our research.

Major points 4:The molecular structure shown in Figure 8 contains inaccuracies, particularly regarding the depiction of entangled chemical bonds, which appear unrealistic. The authors should revise this figure to accurately reflect the molecular design and avoid misleading representations.

Response Major points 4:*We would like to thank you for your detailed review and valuable comments on Figure 8. In response to your concern regarding the inaccurate molecular structure, particularly the issue with the entangled chemical bonds, we have made comprehensive revisions to*

the figure.

The specific modifications are as follows: Re-adjustment of chemical bonds: We carefully examined all the chemical bonds in the figure, particularly those involving complex structures, ensuring they accurately reflect the molecular design. We rearranged the atoms and chemical bonds to eliminate any crossing or entangling issues. To more accurately represent the spatial relationships between molecules, we adjusted the positions and angles of the molecules, thereby eliminating the unreal feeling present in the previous version of the figure. We have regenerated the image based on these modifications.

Once again, we sincerely appreciate your valuable feedback.

Major points 5:All figures in the manuscript are difficult to interpret due to low resolution and insufficient labeling

Response Major points 5:*We would like to thank the reviewer for their valuable feedback on the image quality. We have revised all the images and increased their resolution to the highest quality to ensure that the details are clearly visible. Additionally, for the convenience of review, we have compiled all the modified high-resolution images into a compressed folder and uploaded it. We hope that the new images will improve readability and better showcase our research findings.*

Major points 6:The presentation of the manuscript needs a thorough revision. The results section should be reorganized to highlight the most important findings more clearly, and the conclusion should emphasize the key contributions of the study. The narrative should be streamlined to solidify the connection between the findings and their implications for cancer immunotherapy, avoiding over-speculation in the discussion.

Response Major points 6:*We would like to thank you for your detailed review and valuable feedback on our manuscript. Based on your suggestions, we have made comprehensive revisions to the Results and Conclusion sections and implemented the following adjustments:*

1.Reorganization of the Results Section: We have simplified and reorganized the Results section to highlight the most important findings of the study, particularly the relationship between the different differentiation states of myeloid-derived suppressor cells (MDSCs) and tumor immune evasion. We have streamlined the narrative by reducing excessive details, ensuring a clearer presentation of our key findings.

2.Adjustment of the Conclusion Section: In response to your suggestion, we have strengthened the key contributions of the study and clearly articulated the important role of MDSCs in tumor immune evasion. We have further emphasized the potential of the MDSC-based differentiation feature gene risk prognostic model in predicting patient prognosis and assessing immune therapy responses. Additionally, we have simplified the expression to avoid over-speculation in the discussion, placing more emphasis on the connection between our findings and cancer immunotherapy.

3.Streamlining the Narrative: To enhance the flow of the manuscript, we have simplified certain

parts of the narrative to reduce unnecessary complexity, ensuring a direct connection between our findings and cancer immunotherapy. Specific changes have been made in the following sections of the manuscript: lines 93-106, 114-141, 154-170, 176-197, 259-278, 286-318, and 324-346.

We believe these revisions better highlight the core findings of the study and make the manuscript more concise and impactful. We greatly appreciate your constructive suggestions and look forward to your further feedback.

Comments on the Quality of English Language:The manuscript contains several instances where the phrasing is unclear and the terms are insufficiently explained, making it difficult to fully understand the authors' intended meaning. For example, in the statement: "In various cancer types, including breast, gastrointestinal, and head and neck cancers, researchers have observed elevated expression levels of MDSCs, which appear to be a common immune evasion strategy employed by tumors" the phrase "elevated expression levels of MDSCs" is ambiguous. Since MDSCs are a cell population rather than a gene or protein, it is unclear whether the authors are referring to an increase in the abundance of MDSCs, the upregulation of specific functional markers, or enhanced immunosuppressive activity. Similarly, in the statement: "We identified PMN-MDSCs as the predominant subset in state one based on the expression levels of CD83, while the remaining subset consisted of M-MDSCs," the terms PMN-MDSCs and M-MDSCs are introduced without explanation, leaving the reader unsure of their specific meaning.

Response Comments on the Quality of English Language:*Thank you for your valuable feedback. In response to the two issues you raised, I have made the following revisions:*

1.In the description of MDSC expression levels, I have clarified that it refers to the increased abundance of MDSCs and further elaborated on the significance of this statement (lines 58-62).

2.Regarding the terms PMN-MDSCs and M-MDSCs, I have provided a detailed explanation (lines 130-133) to ensure that readers can clearly understand the meanings of these terms.

Additionally, I have carefully reviewed the manuscript and made corresponding revisions throughout to ensure the language is clearer and more comprehensible, with terms fully explained and used accurately.

Once again, thank you for your feedback. Your suggestions have been instrumental in improving the quality of the manuscript.

For review article

Response to Reviewer 3 Comments

1. Summary

Thank you for giving us the opportunity to revise and resubmit our manuscript titled "Constructing a prognostic model for NSCLC risk based on genes characterising the differentiation of MDSCs." We have carefully considered your comments and suggestions and have supplemented the relevant information accordingly. We hope that everyone will accept our modifications.

**2. Questions for General Evaluation**	**Reviewer's Evaluation**	**Response and Revisions**
Does the introduction provide sufficient background and include all relevant references?	Yes	
Is the research design appropriate?	Yes	
Are the methods adequately described?	Yes	
Are the results clearly presented?	Yes	
Are the conclusions supported by the results?	Yes	

3. Point-by-point response to Comments and Suggestions for Authors

Comment 1:Influence of Tumor Stage and Treatment on the Immune Microenvironment

Is the immune microenvironment in each NSCLC case assumed to be similar, regardless of tumor extension or treatment history? Since NSCLC patients may be under different immunological conditions due to prior therapies, pre-existing immune status, tumor stage, etc., it would be valuable to provide information on the clinical background of the examined tumors.

Response Comment 1: *Thank you for your valuable comments on our study, particularly regarding the impact of tumor staging and treatment history on the immune microenvironment. We fully agree with your viewpoint that different tumor stages and treatment histories can significantly influence the immune microenvironment, especially in NSCLC, where these factors*

may greatly affect the phenotype and function of MDSCs.

In our study, the risk prognostic model we constructed does indeed take into account clinical factors such as tumor staging, and we have demonstrated that this model performs well in prognostic prediction. However, the main focus of this study is on identifying key genes associated with MDSC differentiation and constructing a new risk prognostic model based on these genes. Our aim is to gain a deeper understanding of the important genes in the MDSC differentiation process and their potential impact on NSCLC prognosis through molecular-level analysis.

At the same time, we fully acknowledge the impact of factors such as tumor staging on the immune microenvironment and have reflected on this in our discussion. Although we did not expand on the specific effects of tumor staging and treatment history on the immune microenvironment in this study, we believe that this research provides new perspectives for the in-depth investigation of MDSC-related molecular mechanisms.

Once again, thank you for your valuable suggestions. In the revised manuscript, we will discuss the complexity of the immune microenvironment in more detail and emphasize how factors such as tumor staging may influence MDSC function and differentiation.

Comment 2:Practical Considerations for Therapeutic Decision-Making

From a clinical perspective, making therapeutic decisions based on each patient's immune microenvironment would be highly complex. It may be helpful to propose some form of guidelines for therapeutic selection according to the microimmunological state.

Response Comment 2: *Thank you for your valuable comments on our study, particularly regarding the practical considerations for treatment decisions. We fully agree with your viewpoint that making treatment decisions based on the immune microenvironment of each patient is indeed a complex process, especially considering the heterogeneity of the tumor immune microenvironment and individual patient differences.*

In this study, our focus was on constructing a risk prognostic model associated with MDSC differentiation to gain deeper insights into the immune microenvironment. However, we also recognize that relying solely on one aspect of the immune microenvironment for treatment decisions may not be sufficient. Therefore, in addition to analyzing the immune microenvironment, we have also incorporated methods such as molecular docking and PyMOL to predict potential therapeutic strategies. These methods help us identify key targets related to MDSCs and assess the binding affinity of potential drugs with these targets, thereby providing more scientific evidence for the development of personalized treatment plans.

Although the primary focus of our study is on molecular mechanisms, we believe that combining molecular docking predictions with immune microenvironment analysis can offer valuable insights for future treatment decisions. We agree with your suggestion that the development of a "treatment selection guideline" is an important direction, and future research could further integrate these various pieces of information to form a more comprehensive framework for treatment

decision-making.

Once again, thank you for your valuable suggestions.

For review article

Response to Reviewer 2 Comments

1. Summary

Thank you for giving us the opportunity to revise and resubmit our manuscript titled "Constructing a prognostic model for NSCLC risk based on genes characterising the differentiation of MDSCs." We have carefully considered your comments and suggestions and have supplemented the relevant information accordingly. We hope that everyone will accept our modifications.

**2. Questions for General Evaluation**	**Reviewer's Evaluation**	**Response and Revisions**
Does the introduction provide sufficient background and include all relevant references?	Can be improved	Strengthen the background description, ensure all relevant references are included, and further clarify the motivation and objectives of the study.
Is the research design appropriate?	Can be improved	We have made revisions to some sections to ensure that the design steps and methods are communicated more clearly to the readers.
Are the methods adequately described?	Must be improved	Provide detailed explanations for each analytical step and clarify certain technical details.
Are the results clearly presented?	Must be improved	Simplify the narrative in the results section and enhance the clarity of the figures and data to make them easier to

		understand.
Are the conclusions supported by the results?	Can be improved	Improve the conclusion section to make it more rigorous and directly reflect the research results, while avoiding excessive speculation.

3. Point-by-point response to Comments and Suggestions for Authors

Comments 1:Trajectory Analysis Validation: The authors claim to have validated their trajectory analysis results using pseudotime analysis but fail to specify which algorithm was used for this validation. While both pseudotime analysis and trajectory analysis aim to infer dynamic cellular processes, they differ significantly in their biological interpretations. Pseudotime analysis orders cells along a continuous progression based on transcriptomic similarities, typically following a single linear path. In contrast, trajectory analysis reconstructs full differentiation or transition paths, including branching points that indicate distinct cell fate decisions. The latter is inherently more complex and requires graph-based inference, incorporating pseudotime to measure cell progression within different paths. Given these distinctions, the authors' response does not convincingly justify the use of pseudotime analysis alone to validate trajectory analysis results. A more rigorous validation strategy, potentially incorporating alternative trajectory inference methods, is necessary to strengthen the findings.

Response Comments 1: *Dear Reviewer,Thank you for your thorough review and valuable feedback on our study. We greatly appreciate your comments regarding the validation of trajectory analysis, and we fully agree that pseudotime analysis and trajectory analysis exhibit significant differences in their biological interpretations. Below, we provide a detailed response to your concerns and outline the modifications we have made to address them.*

1.Regarding the specific algorithms used:In our study, we employed the following two algorithms for pseudotime analysis and trajectory inference:Monocle (v2.26.0): Monocle is a pseudotime analysis algorithm that orders cells along a continuous process based on transcriptomic similarity, inferring dynamic changes in cell states. We used Monocle to construct the pseudotemporal trajectories of myeloid-derived suppressor cells (MDSCs) and annotated distinct differentiation states along with their differentially expressed genes. The results from Monocle provided us with a preliminary inference of the developmental trajectories of MDSCs.Slingshot (v1.8.0): Slingshot is a graph-based trajectory inference algorithm that reconstructs cell differentiation paths and identifies branch points and transitional states. We applied Slingshot to further validate the results from Monocle and capture potential branching events during MDSC differentiation. The results from Slingshot not only supported the number and order of states inferred by Monocle but also provided more complex trajectory information, such as key transitional states and multi-fate decisions.By combining the results from Monocle and Slingshot, we implemented a dual-validation strategy, significantly enhancing the reliability of our trajectory analysis. Monocle provided a linear

perspective based on pseudotime ordering, while Slingshot complemented this with graph-based complex trajectory inference. Together, these approaches offer a more comprehensive understanding of the dynamic evolution of MDSCs.

2.Additional clarification on trajectory analysis validation:In our study, we initially used pseudotime analysis (via Monocle) to infer the developmental trajectories of MDSCs. To further validate these trajectories and capture potential branching events, we incorporated Slingshot, a trajectory inference tool, in the revised manuscript. Slingshot reconstructs cell differentiation paths based on dimensionality-reduced data (e.g., t-SNE or UMAP) and identifies key transitional states and branch points, thereby providing more complex trajectory inference results.By integrating the results from Monocle and Slingshot, we observed a high degree of consistency in the differentiation paths and state transitions of MDSCs. For instance, the trajectory predicted by Slingshot not only validated the number and order of states inferred by Monocle but also revealed key transitional states and potential branching events. This dual-validation strategy significantly strengthened our understanding of the dynamic evolution of MDSCs.

3.Future directions for improvement:We agree with your suggestion that additional trajectory inference methods (e.g., PAGA or SCORPIUS) could be incorporated in future studies to further validate our results and enhance their robustness. Additionally, we plan to complement our computational inferences with experimental validation, such as cell fate tracking or functional assays, to provide further support for our findings.

4.Specific revisions in the manuscript:We have revised and supplemented the relevant sections of the manuscript, including the Results section (lines 123-127), figure legends (lines 145-148), and Methods section (lines 604-618), to clarify the use of Monocle and Slingshot and their complementary roles in our analysis.

Once again, we sincerely appreciate your valuable feedback. We believe that these revisions have adequately addressed your concerns and significantly improved the rigor and reliability of our study. Should you have any further questions or suggestions, we would be happy to make additional revisions.

Comments 2: Differential Expression Methodology: The manuscript lacks crucial methodological details regarding the differential expression analysis. Specifically, the authors do not explain: Which statistical method was used to identify differentially expressed genes; How effect sizes were estimated; How p-values were adjusted for multiple testing to control false positives.Without a clear explanation and proper statistical controls, the reported 8,659 differentially expressed genes may include a high number of irrelevant genes, reducing the reliability of the findings. The authors should provide a detailed description of their statistical approach, including the methods used for multiple testing correction (e.g., Benjamini-Hochberg FDR correction), and ensure that thresholds for both effect size and p-value are appropriately set.

Response Comments 2: *Dear Reviewer,Thank you for your valuable feedback. To enhance the transparency of our differential expression analysis and provide sufficient detail, we have revised*

the Methods section (lines 620-641) and the Results section (lines 204-225) in the manuscript to clearly articulate the statistical methods used in our screening of differentially expressed genes (DEGs). Below, we provide a detailed explanation of the supplementary content:

1.Identification of Differentially Expressed Genes:We employed the DESeq2 package for differential expression analysis. DESeq2 is a widely used method based on a negative binomial distribution model, designed to identify DEGs from count data. DESeq2 first normalizes raw data to eliminate technical variations between samples and then estimates the variability of gene expression data using the negative binomial distribution model, calculating differences in expression levels for each gene. This method effectively controls technical noise and enhances statistical power.

2.Effect Size Estimation and Filtering:During the screening of DEGs, we used log2FoldChange as an indicator of effect size. We set a threshold of |log2FoldChange| > 1.5, ensuring that only genes with significant expression changes were selected. This effect size threshold ensures that we focus on genes with biologically meaningful changes, avoiding the inclusion of genes with minimal or no biological significance, thereby improving the reliability and interpretability of our results.

3.Multiple Testing Correction:To address the issue of multiple hypothesis testing and reduce the false discovery rate (FDR), we applied the Benjamini-Hochberg FDR correction to all calculated p-values. The FDR correction is an effective method for controlling the false positive rate by adjusting p-values to account for multiple comparisons. In our analysis, only genes with padj < 0.01 were considered significantly differentially expressed. This correction process effectively controls false positives arising from multiple testing.

4.Reliability of DEG Screening Results:To ensure the reliability of the identified DEGs, we applied multiple criteria for gene filtering, ensuring that the selected genes are biologically significant. During the DESeq2 analysis, we first filtered out low-expression genes and then selected the final DEGs based on adjusted p-values and effect size thresholds. This approach significantly enhanced the reliability of our results. Ultimately, we identified cytokine genes associated with the differentiation states of myeloid-derived suppressor cells (MDSCs) and further validated their biological significance through subsequent statistical analyses.

5.Transparency of Data Analysis:To ensure the transparency and reproducibility of our findings, we have provided a detailed description of each step in the data analysis process in the Methods section, including the specific criteria for DEG screening (e.g., p-value thresholds, effect size, and correction methods). These details will help reviewers and readers understand how we controlled for false positives and ensured that the final selected genes reflect true and biologically meaningful changes.

Through these improvements and additions, we believe we have fully addressed all concerns raised by the reviewer regarding differential expression analysis, further enhancing the transparency and reliability of our study.

Comments 3:Figure Representation: In Figure 8, there are still errors in the depiction of chemical bonds, particularly in panels E and F, where the bonds are inaccurately represented compared to the correct structure documented in the chemical database.

Response Comments 3:*Dear Reviewer,Thank you for your meticulous review and valuable feedback regarding the figures in our manuscript. In response to your comments on the issues with the chemical bond descriptions in Figure 8, particularly the discrepancies in the structures depicted in panels E and F compared to the correct structures recorded in chemical databases, we have carefully revised all relevant images in Figure 8. The updated figures have been incorporated into the revised version of the manuscript.We greatly appreciate your attention to the details in the figures. These revisions have significantly improved the accuracy and credibility of our manuscript.*

Minor Issues 1:The manuscript cites figures using "Figure A" without the figure numbers, making it difficult to locate the corresponding figures

Response Minor Issues 1:*Dear Reviewer,Thank you for your valuable feedback regarding the citation format of figures and tables in our manuscript. In accordance with your suggestions, we have revised all figure and table citations throughout the manuscript. All figures and tables are now labeled with clear numerical identifiers, making it easier for readers to locate the corresponding figures and tables. We have compressed all the figures into a single file named Figure.zip and uploaded it for your review. Please kindly check the updated figures at your convenience.We believe this improvement will significantly enhance the readability and accuracy of the manuscript.*

Minor Issues 2:Figure legends lack sufficient detail. For instance, in Figure 8, the authors should explicitly describe which molecules are depicted in each panel to improve clarity and reader comprehension.

Response Minor Issues 2:*Dear Reviewer,Thank you for your valuable feedback regarding the details of the figure legends. In accordance with your suggestions, we have thoroughly revised the legend for Figure 8(lines 348-352). In the updated legend, we have provided clear descriptions of the molecules depicted in each panel, along with relevant details, to improve clarity and facilitate reader comprehension. We believe this revision will significantly enhance the readability of the figure and help readers better understand the visual content.Once again, we sincerely appreciate your constructive comments.*
